# Morphology and morphometry of *Haemonchus contortu*s exposed to *Gigantochloa apu*s crude aqueous extract

**DOI:** 10.14202/vetworld.2018.921-925

**Published:** 2018-07-14

**Authors:** Budi Purwo Widiarso, Kurniasih Kurniasih, Joko Prastowo, Wisnu Nurcahyo

**Affiliations:** 1 Doctoral Program Parasitology Department, Faculty of Veterinary Medicine, Universitas Gadjah Mada, Yogyakarta 55281, Indonesia; 2 Department of Animal Health, Agriculture Extension College (STPP), Magelang, Jalan Magelang-Kopeng Km 7 Purwosari, Tegalrejo, Magelang, Jawa Tengah, 56192, Indonesia; 3 Department of Pathology, Faculty of Veterinary Medicine, Universitas Gadjah Mada, Bulaksumur, Yogyakarta 55281, Indonesia; 4 Department of Parasitology, Faculty of Veterinary Medicine, Gadjah Mada University, Bulaksumur, Yogyakarta 55281, Indonesia

**Keywords:** bligon goat, crude aqueous extract, *Haemonchus contortus*, morphology, morphometry

## Abstract

**Aim:**

*Haemonchus contortus* is the most pathogenic nematode infesting the digestive tract of goats and sheep worldwide leading to a tremendous loss in a variety of routes. Economic losses due to haemonchosis in subtropic and tropic areas are usually caused by poor weight gain, minimized growth, loss of production, and mortality. The prevalence of haemonchosis in Indonesia is 89.4% in goat, and annual loss achieved 1 million US dollars. This study evaluated *in vitro* effects of *Gigantochloa apus* crude aqueous extract as an anthelmintic on *H. contortus* morphology and morphometry.

**Materials and Methods:**

Bligon goats which are naturally infected were collected from slaughtered goat from local slaughterhouses, namely Besi Sleman. Bligon goat’s abomasum part was carefully examined and transported to the Parasitology Laboratory, University of Gadjah Mada, Yogyakarta. *H. contortus* was obtained from 4 to 6-month-old female goat from slaughterhouses in Yogyakarta area. *H. contortus* was collected from abomasum and put into a Petri dish containing 0.62% water saline. The number of *H. contortus* used for each concentration is 25. *H. contortus* was soaked in each concentration for 4 h. The figure of the parasites or parts of parasites was captured using camera Lucida, and they were measured using both objective micrometer and objective ocular micrometer. All the capturing processes were done with the help of Olympus Digital Camera under Olympus CX21 microscopic. Parasite morphology was identified in morphological and morphometric characters.

**Result:**

Morphology of *H. contortus* revealed the cervical papillae bulge appears unclear shape and anterior end is more tapered. Vulvar flab control is not tapered, but vulvar flab which gets aware of *G. apus* crude aqueous extract looks more pointed. The gubernaculum appears irregular compared to gubernaculum control which tends to be more compact, and the posterior end form appears irregular more than posterior end control. Morphometry study of *H. contortus* indicates that it has a significant difference for body length, body width, cervical papillae, and spicule length in the male.

**Conclusion:**

*G. apus* crude aqueous extract activity revealed morphology change such as cervical papillae, vulvar flab, gubernaculum, posterior end, and reduced morphometry measurement of *H. contortus* adult worms, notably in body length, body width, cervical papillae width, gubernaculum, and spicule length in males and body length, body width, cervical papillae width, and vulva length in females.

## Introduction

*Haemonchus contortus* is the most pathogenic nematode infesting the digestive tract of goats and sheep worldwide. It leads to a tremendous loss in a variety of routes [1]. Poor weight gain, retarded growth, loss of production, and mortality usually cause economic losses because of haemonchosis in subtropic and tropic areas [2,3]. The prevalence of *Haemonchosis* in Indonesia is 89.4% in goat, and annual loss achieved 1 million US dollars [4].

For decades, the use of anthelmintic chemicals for the therapy of gastrointestinal nematode infections in cattle has present of drug resistance. In addition to spending a considerable cost to find new types, anthelmintic also often poses a problem for the safety of food products of animal origin [5]. Anthelmintic resistance is responded by making the discovery of natural substances with low toxicity to reduce the burden of worms in livestock [6]. Many innovations are made to find alternatives to anthelmintic by selecting some plants that contain tannin because it is reported that the substance can reduce the incidence of worm infestation. The development of new anthelmintics suggests that plants containing tannin (Tannin plants) can be considered as potential strategic alternatives for the control of nematode infestation in small ruminants [7]. Plant biological resources have been widely used by breeders and researchers to help to increase the growth of their farms. One source of biological tanninferous plants that can be a new alternative is the use of bamboo leaves to help the farm business [8]. *Gigantochloa apus* crude aqueous extract was made by chopping bamboo leaves into smaller pieces. Chopped apus bamboo leaves were weighed according to the desired weight or concentration, which was 1 g and 10 g for stock solutions. Both chopped apus bamboo leaves are put into separate beakers. The beakers filled with bamboo leaves were then filled with 100 ml of Aquadest. The beakers were then put into an oven with a temperature of 90°C for 15 min [9].

This study evaluated *in vitro* effects of *G. apus* crude aqueous extract as an anthelmintic on *H. contortus* morphology and morphometry.

## Materials and Methods

### Ethical approval

All stages of the research were approved by the Ethical Committee of Gadjah Mada University (number 00118/04/LPPT/IX/2017).

### Tools and materials

The research materials include tools and materials used during the research. The tools used are as follows: Petri dish to observe adult worms’ motility and mortality rate, object glass to make worm preparation, microscope to observe part of the worm, camera Lucida to paint the worm, stopwatch to measure *Haemonchus* killing time submerged by *G. apus* crude aqueous extract, oven to make apus bamboo crude aqueous extract, Erlenmeyer flask to make apus bamboo crude aqueous extract in various dosages, electric scale to measure bamboo leaf weight, surgical scissors to cut off the abomasal line for worms exploring, and the length and body part of *H. contortus* were measured by micro caliper. The materials used are *G. apus* crude aqueous extract, *H. contortus* adult worm, Aquadestilata, ethanol, and 0.62% NaCl. Morphometric test of *H. contortus* uses concentration at 0, 0.1, and 1 mg/ml to find the lowest effective dose of killing the worms as a dose base determination for *in vivo* research.

### Parasites collection

Bligon goats which are naturally infected were collected from slaughtered goat from local slaughterhouses, namely Besi Sleman. Bligon goat’s abomasum part was carefully examined and transported to the Parasitology Laboratory, University of Gadjah Mada, Yogyakarta. *H. contortus* was obtained from 4 to 6-month-old female goat from slaughterhouses in Yogyakarta area. *H. contortus* was collected from abomasum and put into a Petri dish containing 0.62% water saline. The number of *H. contortus* used for each concentration is 25. *H. contortus* was soaked in each concentration for 4 h.

The figure of the parasites or parts of parasites was captured using camera Lucida, and they were measured using both objective micrometer and objective ocular micrometer. All the capturing processes were done with the help of Olympus Digital Camera under Olympus CX21 microscopic. Parasite morphology was identified in morphological and morphometric characters [6].

### G. apus crude aqueous extract

In making 1% *G. apus* crude aqueous extract, according to Daryatmo *et al*. [9], crude aqueous extract of apus bamboo leaves was made by chopping bamboo leaves into smaller pieces. Chopped apus bamboo leaves were weighed according to the desired weight or concentration, which was 1 g and 10 g for stock solutions. Both chopped apus bamboo leaves are put into separate beakers. The beakers filled with bamboo leaves were then filled with 100 ml of Aquadest. The beakers were then put into an oven with a temperature of 90°C for 15 min. The remaining liquid in the beakers was taken and filtered to obtain a concentration of 0.1% and 1% *G. apus* crude aqueous extract.

### Statistical analysis

Morphology of *H. contortus* adult worms at various concentrations was determined by microscopic observation. The differences in morphometry variables of *H. contortus* were recorded and analyzed using SPSS software 16.0.

## Results

### G. apus leaf crude aqueous extract on morphology of female and male H. contortus adult worms

**Figure-1:**
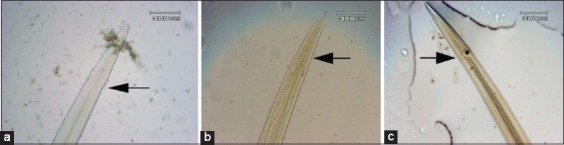
Morphology cervical papillae: (a) Cervical papillae which is not soaked in *Gigantochloa apus* crude aqueous extract; (b) cervical papillae which is soaked in *G. apus* crude aqueous extract 0.1 mg/ml concentration; (c) cervical papillae which is soaked *G. apus* crude aqueous extract 1 mg/ml concentration.

**Figure-2:**
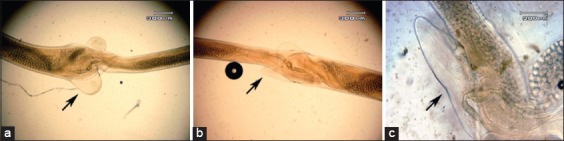
Morphology of vulvar flab: (a) Vulvar flab which is not soaked in *Gigantochloa apus* crude aqueous extract; (b) vulvar flab which is soaked in *G. apus* crude aqueous extract 0.1 mg/ml concentration; (c) vulvar flab which is soaked in *G. apus* crude aqueous extract 1 mg/ml.

**Figure-3:**
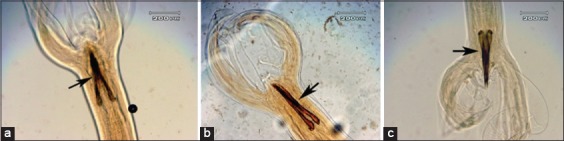
Morphology of gubernaculum: (a) Gubernaculum which is not soaked in *Gigantochloa apus* crude aqueous extract; (b) the gubernaculum which is soaked in *G. apus* crude aqueous extract at a concentration 0.1 mg/ml; (c) gubernaculum which is soaked in *G. apus* crude aaqueous extract with concentration of 1 mg/ml.

**Figure-4:**
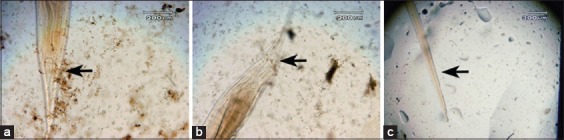
Morphology of posterior end: (a) Posterior end which is not soaked in *Gigantochloa apus* crude aqueous extract; (b) posterior end which is soaked in *G. apus* crude aqueous extract at concentration 0.1 mg/ml; (c) posterior end which is soaked in *G. apus* crude aqueous extract with concentration 1 mg/ml.

Comparative characteristics are shown in Figures-1-4. The body is filiform (slender) tapering toward the anterior end in male and female. The anterior end is relatively wide and blunt. The buccal cavity is small with a conspicuous tooth extending from the dorsal wall. There are no buccal capsules. In addition to transverse striation, longitudinal lines are also present on the body.

Figure-1 shows that cervical papillae in control (a) appear clearly and conspicuous and part of cervical papillae at the concentration of 1 mg/ml (b) and 0.1 mg/ml (c). The anterior end is more tapered, and the cervical papillae bulge appears unclear shape.

Figure-2 observes that vulvar flab control is not tapered (a). Vulvar flab which gets aware of *G. apus* concentration of 0.1 mg/ml and 1 mg/ml crude aqueous extract of *G. apus* looks more pointed. Vulvar flab in concentration of 1 mg/ml is sharpener and cleaner than 0.1 mg/ml concentration.

Figure-3 shows that gubernaculum form exposed to *G. apus* (b/c) appears irregular compared to control gubernaculum which tends to be more compact. Spiculas exposed to *G. apus* appear more budding than control spicula.

Figure-4 shows that the posterior end form exposed to *G. apus* crude aqueous extract at a concentration of 0.1 mg/ml and 1 mg/ml appears irregular (b/c) more than posterior end control.

### Apus bamboo leaf crude aqueous extract on morphometry of female and male H. contortus adult worms

The result reports that bamboo leaf crude aqueous extract affects morphometry of female and male *H. contortus* adult worms such as body length of the worm, body width of the worm, cervical papillae width, spicule length, gubernaculum length, and vulva length (Tables-1 and 2).

**Table-1:** Morphometry of female *H. contortus* adult worms due to the administration of bamboo leaves crude aqueous extract *in vitro*.

Worm morphometry	Control (0%)	0.1 mg/ml *G. apus* crude aqueous extract (mm)	1 mg/ml *G. apus* crude aqueous extract (mm)
Body length	27.25±2.59^a^	26.30±1.75^b^	24.30±1.85^c^
Body width	0.64±0.08^a^	0.48±0.09^b^	0.42±0.07^c^
Cervical papillae width	0.38±0.08^a^	0.36±0.06^a^	0.33±0.08^b^
Vulva length	4.67±0.31^a^	4.12±0.22^a^	3.87±0.67^b^

a,b,c Different superscripts within row indicate significant differences (p<0.05). The number of worms measured for each concentration is 25, *H. contortus=Haemonchus contortus, G. apus=Gigantochloa apus*

**Table-2:** Morphometry of male *H. contortus* adult worms due to the administration of bamboo leaves crude aqueous extract.

Worm morphometry	Control (0%)	0.1 mg/ml *G. apus* crude aqueous extract (mm)	1 mg/ml *G. apus* crude aqueous extract(mm)
Body length	17.25±0.79^a^	16.30±0.71^b^	14.30±1.85^c^
Body width	0.29±0.06^a^	0.22±0.05^b^	0.20±0.04^b^
Cervical papillae width	0.44±0.03^a^	0.41±0.06^a^	0.38±0.08^b^
Spicula length	0.52±0.01^a^	0.42±0.22^a^	0.38±0.07^b^
Gubernaculum length	0.22±0.02^a^	0.20±0.01^a^	0.18±0.03^a^

a,b,c Different superscripts within row indicate significant differences (p<0.05). The number of worms measured for each concentration is 25, *H. contortus=Haemonchus contortus, G. apus=Gigantochloa apus*

Morphometry of female *H. contortus* produces a body length and body width of 27.25±2.59 mm and 0.64±0.08, respectively. Vulva is located in the posterior third of the body at a distance of about 4.67±0.3 1 mm from the posterior end. The vulvular lips are inconspicuous, but a linguiform process is invariably present. Vulva is covered with valves.

Morphometry of male *H. contortus* produces a body length and body width of 17.25±0.79 mm and 0.29±0.06, respectively. The tail end bears a bursa. The bursa consists of three lobes, two large lateral lobes, and poorly developed dorsal lobe. Dorsal ray is asymmetrical and bifurcated. Externo-dorsal ray is thin and long. Lateral rays arise from a common trunk, and ventral rays are fused proximally and separated dorsally.

The result of microscopic observation on *H. contortus* adult worms indicates that it has a significant difference in body length, body width, cervical papillae, and spicule length in male. There is no significant difference for gubernaculum length. *G. apus* crude aqueous extract at concentration of 0.1 mg/ml and 1 mg/ml is able to shorten the body length and spicule length. *G. apus* crude aqueous extract was decreased the body width in male adult worms.

## Discussion

From the last study about *H. contortus* morphology [1], the shape is observed to be similar to the morphology of *H. contortus* with Reyaz [10] characters including color, total length, maximum width, spicule length, vulva length, gubernaculum length, and cervical papillae width.

Based on Table-2, it can be determined that there is a significant difference in the body width and body length of female *H. contortus* adult worms among the concentration of 0.1 mg/ml, 1 mg/ml, and 0 mg/ml (control). On the width of cervical papillae, there is no significant difference between the concentration of 0.1 mg/ml and 0 mg/ml (control), but at the concentration of 1 mg/ml, *G. apus* crude aqueous extract has a significant difference to the control. The length of vulva has no significant difference at the concentration of 0.1 mg/ml to the control, but the concentration of 1 mg/ml has a significant difference to the control. The concentration of 1 mg/ml also has a significant difference to the concentration of 0.1 mg/ml in *G. apus* crude aqueous extract. The cuticle’s damage caused by tannins contained in apus bamboo leaves can decrease the length of the worm’s body, the body width, the width of cervical papillae, and the length of vulva. The tannin disturbs the physiology process of nematodes by binding nematode protein directly and subsequently [11].

Preliminary phytochemical screening of *G. apus* revealed saponin, alkaloid, flavonoid, and tannin. Tannin in *G. apus* can influence both directly and indirectly with adult worms. Direct reaction happens by attaching their cuticle and causing distress, while indirectly reaction happens by improving protein nutrition [12].

Tannins on apus bamboo leaves play a role in binding proteins and turning nematode walls into inactivity and killing them as reported by Hoste *et al*. [13]. In addition, Molan *et al*. [14] proved that condensed tannins may have different effects on ruminants when consumed by the growth of adult worms and larvae. Calvin *et al*. [15] showed that condensed tannins have anthelmintic activity with varied possible mechanism of actions, most especially astringent property.

Microscopic observation on *H. contortus* morphometry indicates that it has a significant difference about body length, body width, cervical papillae width, gubernaculum, and spicule length in male worms (Table-2). *G. apus* crude aqueous extract concentrations of 1 mg/ml and 0.1 mg/ml were able to shorten the body length of male adult worms. Both the concentrations of bamboo leaf crude aqueous extract, 0.1 mg/ml and 1 mg/ml, have a significant difference to the control, especially in the body length, cervical papillae width, and spicule length. The length of spicules has a significant difference between *G. apus* crude aqueous extract at the concentration of 1 mg/ml (0.38±0.07) versus 0.1 mg/ml (0.42%±0.22) and at the concentration of 1 mg/ml versus control (0.52±0.01), but there was no significant difference between the length of spicules of worm exposed to 0.1 mg/ml concentration and those of the control. The width of cervical papillae in male *H. contortus* adult worms differed significantly between the dose of 1 mg/ml *G. apus* crude aqueous extract (0.38±0.08) and the dose of 0.1 mg/ml (0.41±0.06), as well as between the dose of 1 mg/ml and the control (0.44±0.03), but there was no significant difference between 0.1 mg/ml dose and the control on width of cervical papillae. The presence of many morphometric differences between different doses and control may be due to the effect of tannins in *G. apus* crude aqueous extract that can damage the adult worm’s cuticle, interfering with the digestion process. The cuticle change with the longitudinal and transverse wrinkles after *in vitro* exposure to *Biophytum persianum* rich in condensed tannin on *H. contortus* was evaluated by Sambodo *et al*. [16] crude extract. The wrinkles in the cuticle and anterior end of *H. contortus* were also observed by Martinez-Ortiz-de-Montellano *et al*. [17]. The cuticle reveals the form of adult worms and also involved in its motility and the exchanges with the parasite environment, including the metabolic exchanges with the local environment in the digestion tract of the host [1].

## Conclusion

*G. apus* crude aqueous extract activity revealed morphology change and reduced morphometry measurement of *H. contortus* adult worms, notably in body length, body width, cervical papillae width, gubernaculum, and spicule length in males and body length, body width, cervical papillae width, and vulva length in females.

## Authors’ Contributions

The research was determined, managed, and supervised by KK. BPW took samples and recorded samples and sample analysis. WN, BPW, and KK arranged, analyzed, and wrote the report. JP worked overall observation of the experiment and the manuscript writing. All authors read and approved the final manuscript.
